# Measuring child coping in times of societal crises: Pilot development, reliability, as well as mental health and meaning mindset convergent validity of the children’s crisis coping scale (3Cs)

**DOI:** 10.3389/fpsyg.2022.947507

**Published:** 2022-11-09

**Authors:** Laura L. Armstrong, Catherine L. Potter

**Affiliations:** School of Counselling, Psychotherapy and Spirituality, Saint Paul University, Ottawa, ON, Canada

**Keywords:** measure development (psychometrics), child mental health, meaning, meaning - centered coping, second wave positive psychology

## Abstract

To date, there are no brief child self-report coping measures for the pandemic and other major societal events resulting in social or learning disruptions for children. Ignoring the voice of children can ultimately result in programs or services that fail to meet their needs. Thus, a child self-report measure called the 3Cs (Children’s Crisis Coping) was developed and underwent pilot evaluation. This measure was designed in collaboration with key stakeholders using a Knowledge Translation-Integrated development framework. Some of the primary concerns that were relevant in the literature for the development of a pandemic coping measure included stress, worries, loneliness, and unpredictable school changes. The completed 4-item measure, grounded in these concerns, demonstrated good internal consistency reliability, as well as convergent validity with mental health and meaning mindset. A Second Wave Positive Psychology framework is presented concerning a spiritual concept called “meaning mindset” and it’s association with positive societal crisis coping (i.e., pandemic coping in the present study).

## Introduction

At any given point in time before the current global pandemic, 20% of children and youth in North America experienced significant emotional, behavioural, or social challenges (Liratni and Pry, 2011; Kroesbergen et al., 2016; Pilarinos and Solomon, 2017). During the COVID-19 pandemic, however, over *half* of parents reported mental health symptoms in their children (Kar et al., 2020; Shah et al., 2020). Specifically, depressive symptoms, anxiety, contamination obsessions, family well-being challenges, eating disorders, and behavioural concerns have emerged for children during the pandemic (Fegert et al., 2020; Fineberg et al., 2020). Without community-based mental health promotion approaches, such concerns may hinder positive development, personal life trajectory, academic success, and inhibit children from meeting their potential (Pilarinos and Solomon, 2017). In addition to mental health promotion interventions, it is important to be able to measure the effectiveness of such interventions through assessing child coping with the pandemic.

To date, there are no existing, brief pandemic-specific self-report coping measures to assess younger child well-being. In fact, there are no brief child self-report measures for assessing coping with social and learning disruptions due to societal crises (e.g., war, famine, refugee situations, natural or other disasters that destroy infrastructure important to children, etc.). Regarding the current pandemic, most research has explored well-being of younger elementary-age children during the pandemic from a parental perspective. In general, “child friendly” self-report mental health measures for younger children are less common than parent-report measures, with most mental health measures aimed at older children or adolescents (Riley, 2004; Barblett and Maloney, 2010). However, research from both North American and Europe suggests that children are capable of reporting on their own well-being as young as 6 years of age (Riley, 2004; Husky et al., 2018). Where self-reported measures of child well-being do exist along with parental measures, there tends to be low interrater reliability (Riley, 2004; Barblett and Maloney, 2010; Gough Kenyon et al., 2021). Specifically, the parent–child agreement tends to be measured around 20%, similar to the adolescent-parent interrater agreement rates (Riley, 2004; Barblett and Maloney, 2010; Gough Kenyon et al., 2021). Given these discrepancies, excluding child self-report on their own mental health and well-being for programs or services affecting them can ultimately result in services that do not meet child needs and can lead to inappropriate outcomes (Amsden and VanWynsberghe, 2005; Commissioner for Children and Young People, 2016). Including the voice of children is critical, as international policy has stressed the relevance of engaging children in shared decision-making for health services affecting them (Deighton et al., 2014).

To date, where COVID research to date has included child self-report, general mental health measures for children were typically used, rather than pandemic-specific or societal disruption crisis coping measures. One longer measure of COVID-specific coping (i.e., the PICS measure produced by a British Columbia children’s hospital team), completed by children as young as eight-years-old was developed, but administration time of this measure is approximately 20 to 30 min. Such a long administration time may render it potentially inappropriate to use in program evaluations of interventions to address child coping with the pandemic. Thus, it seems imperative that a brief pandemic-specific coping measure, or one that can be used to assess child coping with societal disruption crises beyond the current pandemic, be developed. Such a measure may allow for the effective evaluation of mental health promotion programming or help provide a brief, potentially wider-scale, epidemiological snapshot of child coping. Further, such an evaluation measure would also be useful beyond the current pandemic for any future pandemics or other lockdown or isolating situations that may emerge (e.g., refugee situations). Some of the key concerns that seem important to measure regarding pandemic coping associated with isolation include stress, worries, loneliness, unpredictable school changes, and family coping (Abawi et al., 2020; Clemens et al., 2020; Cost et al., 2021; Mochida et al., 2021; Ullah et al., 2021). Associations between COVID coping with mental health and meaning mindset, a Second Wave Positive Psychology spiritual concept noted below, may also be relevant.

## Child stress and the COVID-19 pandemic

Measuring child stress is seen as critical during the COVID-19 pandemic, as higher levels of perceived child stress (parent-reported and older child self-reported) is predictive of deterioration in all internalizing and externalizing mental health domains (Cost et al., 2021). Thus, if researchers want to explore whether mental health promotion programming is helpful in the shorter-term and predictive of longer-term mental health, it may be important to first explore child coping with stress. During the COVID-19 pandemic, most perceived stress for young people appeared to emerge in response to the disruption of their social life with friends, coaches, or extended family and disruption in meaningful extracurricular or leisure activities, uncertainty about the length of the pandemic and other unknowns, worries about things such as infection, as well as stress in response to distressing news about the pandemic (Mohler-Kuo et al., 2021).

## Child worries and the COVID-19 pandemic

Compared to pre-pandemic times, researchers have found increased levels of worrying in children during the pandemic (Abawi et al., 2020). In fact, health-related fear is normative in the context of an abnormal global situation (Haig-Ferguson et al., 2021). Specifically, in response to an unprecedented, unpredictable, and uncontrollable pandemic situation, if proportionate and adaptive, some worry can motivate helpful precautionary health behaviours like mask-wearing, hand-washing, physical distancing, and vaccination (Taylor, 2019). However, worrying falls along a continuum, with some children experiencing intense worries that affect their everyday functioning (Haig-Ferguson et al., 2021). Avoidance and reassurance-seeking with parents inadvertently colluding with child worries serve as unhelpful coping mechanisms that can maintain or worsen child worries (Haig-Ferguson et al., 2021). Further, reported worries during the pandemic were higher in children whose parents were essential workers than in children whose parents were at home with them, even if a parent had been laid-off work (de Avila et al., 2020). Due to the challenges with worries facing children during the pandemic, assessing child coping with worries and teaching strategies to promote positive coping seem relevant during this time.

## Child loneliness and the COVID-19 pandemic

Given the nature of the transmission of COVID-19, physical distancing was the backbone of prevention protocols during the pandemic. Separation from the typical wider support networks that children have has led to loneliness during the pandemic (Ullah et al., 2021). A child’s sense of loneliness during the pandemic is associated with stress and worries (Cost et al., 2021). The experience of loneliness also appears to have led to difficulties with child identity, given that social cues help to foster identity (Ullah et al., 2021). Further, loneliness during the pandemic is associated with mental illness symptoms or addictive behaviours (Cooper et al., 2021; Sarıalioğlu et al., 2022). Even though socializing virtually was recommended by many health professionals, the more time young people spent texting or otherwise socializing only *via* the Internet, the more mental health or addictive symptoms they reported (Cooper et al., 2021; Sarıalioğlu et al., 2022). Given that loneliness is associated with a number of challenges, it seems relevant to explore child coping with loneliness. One protective factor that leads to good coping with lonely feelings is having a positive relationship with one’s parents (Cooper et al., 2021).

## Child coping with unpredictable school changes and the COVID-19 pandemic

Across many parts of the world, during the pandemic, children have had significant disruption in their academic experience. For both short and longer periods of time, many children received home schooling or virtual schooling. During such times, children often have had to be supported or even taught by their parents or caregivers (de Avila et al., 2020). Research has demonstrated that some children were able to cope with the unpredictability and quick pivot to school at home and then back to in-person learning or vice versa (Clemens et al., 2020). For some children, being at home in a quieter environment, with the structure and support from their parents, away from bullies or social exclusion, for example, may have allowed for such children to thrive (Clemens et al., 2020). However, other children were mildly aversely affected by these school disruptions, unable to practice their social skills or handle being away from learning resources (Clemens et al., 2020). Further, other children had particular difficulty coping with rapid changes or from being away from a school “safe haven” (Clemens et al., 2020). In fact, school closures have disproportionally affected children’s well-being, compared to the well-being of many other age groups, and have widened the chasm between socioeconomically advantaged and disadvantaged children (Munir, 2021). Overall, school disruptions have increased children’s level of stress, worries, and sense of isolation, which has been difficult for many young people (Munir, 2021).

## Family coping and the COVID-19 pandemic

Considering the challenges associated with changes in the daily family life of children should not be underestimated (de Avila et al., 2020). Difficulties with family members’ health and emotional states are inversely associated with child well-being (de Avila et al., 2020). In particular, parental stress during the pandemic has been found to be associated with child stress (Mochida et al., 2021). Family psychosocial stress has in itself been a “contagion” during the pandemic for children (Liu and Doan, 2020). During school and community lockdowns, families had to navigate remote work without childcare, essential work without the usual support network, educate children at home, and also help family members prevent disease transmission (Liu and Doan, 2020): All of these situations can be stressful for families. Further, chronic stress can inhibit rational thought and typical positive coping strategies (e.g., going for a family walk; Liu and Doan, 2020). In addition, loneliness and stress during the pandemic for young people are inversely associated with family coping and a positive parent–child relationship (Janssens et al., 2021). Therefore, it seems that, in addition to child coping with stress, worries, loneliness, and school changes, family coping is another area that may be important to explore during this time period that appears to affect child well-being.

## Positive pandemic coping: Association with mental health and meaning mindset

Research suggests that poor mental health is associated with the challenges noted above (e.g., Abawi et al., 2020; Clemens et al., 2020; de Avila et al., 2020; Cooper et al., 2021; Cost et al., 2021; Mochida et al., 2021; Munir, 2021). Therefore, if young people are positively coping with the challenges associated with the pandemic, they should also exhibit good internalizing and externalizing mental health. Further, “meaning mindset,” a Second Wave Positive Psychology spiritual concept (SWPP; Armstrong et al., 2018), is associated with positive coping (Armstrong et al., 2018; Parrot et al., 2021). SWPP builds on both Logotherapy, a spiritual meaning-oriented approach (Frankl, 1986) and Positive Psychology. SWPP recognizes that, for every challenge—such as the pandemic—there is also something good that may emerge–such as spending valued family time together (Wong, 2011; Wong and Worth, 2017). In SWPP, challenges can be transformed or channeled in a positive manner (Ivtzan et al., 2016; Wong, 2017). Positive motivation and meaningful growth can also be derived because of challenges (Ivtzan et al., 2016; Wong, 2017). Specifically, if difficulties are perceived as opportunities for growth, creativity, or connectedness, then flourishing may occur (Wong and Worth, 2017). Further, difficult experiences can be transformative, allowing for a person to delve wider or deeper into their personal resources (Wong and Worth, 2017). For children, however, there is little guidance in the literature as to how a child may transform a difficult experience, such as a pandemic, into an experience that is meaningful. Despite that a sense of meaning is a positive predictor of COVID coping (Arslan and Yildirim, 2021), there is no roadmap in the literature as to how children in particular may achieve meaning and potentially positively cope with the pandemic. Meaning, as the spiritual aspect of personhood (Frankl, 1986), may be particularly important during difficult times (Wong, 2017). One concept in the literature for children, notably “meaning mindset,” was designed to map onto Frankl’s (1986) Logotherapy concepts and may provide a roadmap to meaningful spiritual growth. More specifically, meaning mindset includes:

1. *Agency over thoughts and behaviours*: The belief that one is capable of, and responsible for, choosing their attitudes and actions, which can in turn regulate their feelings. A person can choose their attitude under almost any circumstance (Frankl, 1986);
2. *Positive self-concept*: Building on growth mindset (Dweck, 2015), positive self-concept within the concept of meaning mindset is the belief that one is a person of worth, as well as capable of achieving their goals though hard work, using good strategies, and seeking help from others, when needed. Having a positive self-concept allows a person to move beyond ‘survival mode’ to perceive meaning in everyday life (Van Tongeren and Green, 2010);
3. *Openness to Experience*: This concept involves an attitude of curiosity and a desire to learn new things. It involves the desire to perceive one’s own and other’s feelings and to be mindfully present in enjoyable activities (e.g., in nature, while engaged in a leisure or extracurricular pursuit, connection to the Divine, or connection with others). Openness allows for a perception of meaning in emotional connection with others, in learning and in creative pursuits, and in appreciation of experiences through moments of awe, flow, or gratitude (Frankl, 1986; Shantall, 1989; Stoddard et al., 2011), and
4. *Hope for the Future*: This concept involves an anticipation of a future that is good, even in spite of difficult current circumstances, and a sense of the possible: A person who has a ‘why’ or a ‘what for’ can often bear almost any ‘now’ (Frankl, 1986; Nietzsche 1889, 2003; Stoddard et al., 2011).

Developing a meaning mindset is strongly associated with lower levels of mental illness and greater levels of well-being in children, youth, and adults (Frankl, 1986; St. John, E., 2017; Wong, 2017; Armstrong et al., 2018). According to a R.E.A.L. ([Rational Emotive Attachment Logotherapy], Armstrong et al., 2018; Parrot et al., 2021; Watt, 2020) perspective, which is a SWPP approach, these four components of meaning mindset—agency, self-concept, openness, and hope—can be cultivated in the following ways, represented by the acronym C.H.A.N.G.E.:

- *Challenge stinking thinking*. Aid children and others in evaluating their thoughts: Is my thought true (evidence for and against)? Can I ask someone for more information or otherwise find more information?
- *“I’m so scared that I’m going to get COVID and die.” What’s the likelihood of that happening? Seek more information when uncertain, such as finding out from a parent or trusted adult that it is rare for children to be severely affected by COVID.*
- *“I cannot do anything right.” What do I like about what I did and what would I do differently next time? Have there been times when I have been good at this? What are some things I am good at?*
- ***H**ealthy actions*. Help children and others to schedule feel-good activities, to set goals and create an action plan to follow them. Recognize predictable triggers for challenges and use calming strategies in these circumstances or problem-solving skills to address the challenges.
- *Can create a plan to use feel good activities such as listening to music, exercising, having a hot drink, talking to a friend (virtually or in person), doing a creative activity (*etc.*) to feel better if stressed, worried, sad, angry, or feeling another strong feeling. Other calm down activities, such as meditation, prayer, and relaxation exercises (imagery, diaphragmatic breathing, progressive muscle relaxation) may also be helpful.*
- *Making regular routines even in the face of major life changes or challenges and scheduling activities to look forward to can be helpful actions to regulate feelings.*
- *In any given person, look for situations that predictably seem to trigger strong feelings. Problem-solve potential ways of managing those situations when the child is calm (*e.g.*, can use collaborative problem-solving,* Greene and Ablon, 2006*). People cannot be reasoned with when experiencing strong feelings: Calm down activities or redirecting a younger person to the next fun thing can be helpful instead in these moments. For example, if it is hard to get a child out the door in the mornings, talk about the fun game you will play with them on the way to school to aim toward a new, fun thing.*
- *Accept circumstance.* Explore issues such as, if I cannot change my circumstances, then I am forced to change myself (Frankl, 1986). Is there some small thing that I can do to help in this situation (moving from disempowerment to empowerment)? Cultivate a “me to we” environment.
- *The fact that there is a pandemic cannot be changed. However, children can do things to help in this situation: They can wear mask or get vaccinated to protect others (giving to the world;* Frankl*,*^ 1986^), *as well as to protect themselves. Further, creating or doing things for others helps to build a sense of meaning.*
- *Need for belonging and self*-*compassion.* Relationships are crucial to defining one’s self-identity, sense of worth, and are a critical pathway to experiencing meaning. Building social literacy skills, such as perspective-taking skills, are important to successful relationships. Self-compassion skills are also critical for a positive self-concept. Having valued relationships (e.g., a warm parent, a trusted adult to turn to, or a close friend) builds confidence and self-esteem.
- *Perspective-taking: What’s a situation in which someone might feel scared? Why might a person feel scared in that situation? What’s a different way that someone could feel in that same situation? Why might they feel differently?*
- *Self-compassion: Practice positive self-talk. What would you say to a friend who messed up or is being hard on themselves? Can you say these things to yourself? What are some fun or relaxing activities that you can do to take care of yourself when you are feeling bad about yourself or something that you did or said?*
- *Gratitude*. Under difficult circumstances a hopeless tunnel vision arises when thoughts spiral from “something is bad” to “everything is bad.” Practicing gratitude can be helpful to break the “everything is bad” attitude and build hope for the future.
- *Set a gratitude routine (*e.g.*, when brushing teeth at night): What are three things, even small things, that I am grateful for today?* E.g.*, I had a warm meal, someone said hello to me, my boots were warm in the cold outdoor air.*
- *Emotional language*. With children and others, it is important to notice and acknowledge feelings in order to enhance their emotional literacy. All feelings are helpful alarm bells for action (safety or questioning thoughts) or potential indicators of the experience of meaning. Emotional literacy is, therefore, a key foundation for openness to experience or for recognizing that a moment may have arisen for challenging unhelpful thoughts.
- *Notice feelings.*
- *What is going on for me right now? I’m feeling scared. Is there a real threat in the environment? Do I have to take action to protect myself? Is it a thought causing the scared feeling? If it’s a thought, then I need to question that thought (see above).*
- *What’s going on for me right now? I’m noticing a pleasurable feeling. I’m enjoying being outside in the forest. This is, therefore, a meaningful moment.*

As noted previously, some children have been resilient or have even thrived during the pandemic, while others have coped poorly. Some research with adults may explain a key pathway to positive coping. Specifically, for adults, a sense of meaning is a key predictor of positive COVID coping (Arslan and Yildirim, 2021). Given the strong association between child mental health and meaning mindset (Parrot et al., 2021), a measure of positive COVID coping should demonstrate convergent validity if it is associated with both of child mental health and meaning mindset.

## Knowledge translation-integrated measure development

### Methodological approach

Development of the current Children’s Crisis Coping (3Cs) scale involved a Knowledge Translation-Integrated (KTI) development (Armstrong et al., 2018). KTI measure development is a participatory methodological approach used to engage participants who would be affected by the survey as potential knowledge-users and as knowledge creators (Armstrong et al., 2018). The aim of a KTI approach is to promote the scientific utility standards, including credibility, acceptability, sustainability, and feasibility (Judd et al., 2001).

Measure *credibility*, as per the standards of the American Psychological Association (1985), involves a demonstration of internal consistency reliability, construct validity, and criterion-related validity. Further, the items should be content and face valid and fit the appropriate literature-based domains of interest (Trochim et al., 2016). Measure *acceptability* involves an assessment of the clarity of the items. More specifically, to be acceptable, for a child survey, items must be “child friendly,” being understood and answered correctly by the child respondents (Cohen et al., 1995; Brancato et al., 2006). Measure *sustainability* involves satisfaction in measure completion by participants (e.g., it is not frustrating to complete), and people who might use the measure express an interest in its use (International Organization for Standardization, 2017). Measure *feasibility* is represented by the average time of questionnaire completion, proportion of missing values, and whether such values are missing at random, and the response rate (Bouwmans et al., 2013).

### Purpose

The purpose of the current study was to develop and validate an adaptable, brief child societal disruption crisis-specific coping questionnaire (pandemic-specific in the current study) using a KTI approach. Specifically, child mental health service providers and parents were consulted to provide feedback on the survey design. Children were also consulted for feedback on credibility, acceptability, sustainability, and feasibility.

### Research questions

Using a KTI approach to measure development the research questions are as follows:

#### Credibility

Is the measure perceived by knowledge-users to exhibit content and face validity: Do knowledge-users express that the questionnaire appears to appropriately encompass literature-based domains representing child COVID-coping? Is the measure internally consistent and does it display construct validity and criterion-related validity? More specifically, is the survey associated with measures of internalizing and externalizing mental health, as well as meaning mindset?

#### Acceptability

Are all items understood and answered correctly by children? Are children satisfied with the measure?

#### Sustainability

Do parents or mental health professionals suggest changing anything in order to enhance sustainability or use of the measure in the future?

#### Feasibility

What is the response rate, how long does it take to complete the questionnaire, and what is the proportion of missing values yielded by administration?

## Methodology

### Participants and procedure

Child participants (*N* = 86 English; *N* = 25 French) were recruited for an 8-week virtual mental health promotion program for elementary school children ages 6 to 12 aimed at enhancing mental health and meaning in daily life. This program was offered in English and French in a major metropolitan region in Canada. It was offered online from July to August 2021, just after schools in the region had experienced a period of virtual learning during a pandemic wave until the end of June 2021. Recruitment occurred through advertisements sent to school boards, as well as through community centres providing services to French families, as well as those providing services to anglophone families. Participants also volunteered to take part in the validation of the 3Cs in English and French. The survey was originally designed in English but was translated and then back translated to develop the French version. Prior to participation in the larger 3Cs evaluation, an initial qualitative e-survey group of five children, their parents, and three mental health practitioners evaluated the content of the English and French surveys for face validity, content domain validity, satisfaction with the measure, and they recommended potential changes. The evaluation of the 3Cs questionnaire was added to our ethical approval for a larger study (Potter, 2022). For the larger study, it became clear that a brief self-report COVID coping measure appeared unavailable to evaluate our own mental health promotion program, so this necessitated its development.

To assess credibility for item generation, child mental health practitioners who are also researchers were asked whether the survey appears to encompasses literature-based domains representing child COVID-coping. To further assess credibility through pilot validation, the survey was hosted by SurveyMonkey, an encrypted and secure website with firewalls that meets Canadian data security standards. Parents completed the online consent form. Correlational statistics were carried out to determine if the survey was associated with measures of internalizing and externalizing mental health, as well as meaning mindset.

Based on the collaborative development with the mental health practitioners, parents and children, to assess item acceptability, these reviewers were consulted regarding item wording. Specifically, they were asked whether children would find each item understandable and whether they perceive that the items may be answered correctly by children. Specifically, the initial five children were asked “Did you understand all of the questions?” Yes/no. “If No, which ones would be hard for kids your age to understand? (Check all that might be tricky to understand).” “Is there a way that we might write this question differently so that it would be better for kids to understand?” (Space to write a suggestion). Children also were given the opportunity to express if any items might lead to frustration in survey completion. Regarding statistical analyses, internal consistency reliability statistics of the pilot data were carried out to determine if certain items appeared to be less reliable, and thus perhaps may be less clear. Poor item reliability could suggest if items should be eliminated or otherwise reworded.

To assess sustainability, for the initial five children to participate in survey completion, they were asked about satisfaction with the measure and whether anything could be changed to make the measure more “fun” to complete. The mental health practitioners were also consulted regarding their thoughts on whether they would be likely to use this measure in their research or in their clinical work.

To assess feasibility, e-consultation on survey length was conducted with the initial mental health practitioners, five children, and their parents. They were asked whether they perceived the measure to be an appropriate length. Through online survey statistics, average completion time was also recorded. Further, response rates and missing values analyses were conducted.

### Measures

#### 3CS

The initial proposed Children’s Crisis Coping Survey was a 5-item measure assessing the domains of perceived ability to cope with stress, worries, feelings of loneliness, and possible school changes, as well as the child’s perception of their family members’ ability to cope with stress during the pandemic. All of the items were worded in such a way that these scenarios (e.g., school changes) were hypothetical and children were asked to reflect on their perceived ability to cope with these possibilities. Specifically, “Imagine that you were in the following situations. Even if you did not have these experiences, think about how you might cope with them (move the slider).” The sliding scale ranged from 0 to 10, from “not at all able to help myself” to “Completely able to help myself.” The items included:

- If you were feeling stressed about the [pandemic],^1^ you would be able to help yourself feel less stressed.
- If you were feeling worried about things during the [pandemic], you would be able to help yourself feel less worried.
- If you were feeling lonely during the [pandemic], you would be able to help yourself feel a little better.
- If you were feeling bothered by school changes during the [pandemic], you would be able to help yourself feel less bothered.
- If your family members were to feel stressed during the [pandemic], they seemed to know what to do to help themselves feel less stressed.

#### ISA

The Interactive Symptom Assessment (Armstrong and Watt, 2020) was used to assess convergent validity with the 3Cs. The 12-item ISA (short form) is self-report measure of well-being and mental illness domains in children including: mood and anxiety symptoms, obsessions and compulsions, behavioural symptom (attention deficits, conduct concerns), and self-esteem (social self-esteem, body image, and satisfaction with accomplishments). Item scores range from 0 to 10, and children can “click” their agreement level under a particular character (Caucasian 6-year-old girl or East Indian 12-year-old boy). Specifically, following a video clip visual demonstration for each item, still images of the characters appear on screen with a button slider underneath. For example, “Isa felt good about the friends in her life this week. Eibe did not feel good about the friends in his life this week.” If a child was completely like ISA this week for this item, this child is instructed by the video to move the slider to the far end under ISA (score of 10). If a child was somewhat like ISA this week for this item, then this child would click or slide the slider part way down the line under the image of ISA. If they perceive themselves to be more like Eibe this week, then they would click or move the slider under Eibe instead. For children who are able to read, the item text and instruction are also provided with each video recording. Especially for test–retest or other repeated measure use, children choose to read the text to respond to the items, allowing for more rapid survey completion than re-watching the videos. The internal consistency reliability of this measure is.83 (Armstrong and Watt, 2020).

#### ChIP-I

The Child Identity and Meaning Questionnaire-Interactive (Armstrong et al., 2019) was also used to validate the 3Cs. The ChIP-I (short form) is a 12-item self-report measure of meaning mindset in children including: choice and responsibility over thoughts, feelings, and behavior, positive self-concept, hope for the future, and openness to experience. Scores for each item range from 0 to 10, wherein children can “click” their agreement level under a particular character (Caucasian 6-year-old girl or East Indian 12-year-old boy) for each item. Following each video clip presenting a visual demonstration of each item, still images of the characters appear on screen with a button slider underneath. For example:

When Chip has a difficult feeling like sadness, fear, or anger, he finds it easy to think about things to feel a bit better. //When Ceira has a difficult feeling like sadness, fear, or anger, she finds it hard to think about something to feel a bit better.

If a child was completely like Chip for this item, this child was verbally instructed in the video clip to move the slider to the far end under Chip (score of 10). If a child was a bit like Chip this week for this item, then this child would click part way down the line under the picture of Chip. If they were more like Ceira, then they would be instructed to move the slider under Ceira instead. For children who can read, the item text is also provided under each video recording. The internal consistency reliability of this measure is 0.81 (Armstrong et al., 2019). This measure has been demonstrated in the literature to be a reliable and valid measure of everyday coping, predictive of positive mental health (Armstrong et al., 2019).

## Results

### Credibility: Item generation

In response to questions regarding perceptions of whether the measure appropriately covers literature-based domains representing child COVID coping, the mental health practitioners stated that the items appeared to adequately reflect the literature and that the items displayed good face validity. Further, the translation and back translation of the French version was assessed by the initial small group of participants to display good face validity and accurately reflect the English item intensions.

### Factor analysis

The factorability of the five 3Cs items was examined, as the larger sample size (*N* = 86 English; *N* = 25 French) met criteria to conduct a Factor Analysis. Correlations and descriptive statistics can be found in Table 1. It was observed that four of the five items correlated at least.5 with at least one other item, suggesting reasonable factorability. Secondly, the Kaiser-Mayer-Olkin (KMO) measure of sampling adequacy was.75, above the commonly recommended value of.6. Further, Bartlett’s test of sphericity was significant [χ^2^(10) = 176.91, *p* < 0.001]. Finally, the communalities were all above.6, further suggesting that each item shared some common variance with another item. Given these overall indicators, factor analysis was deemed to be suitable for all five items.

**Table 1:** Descriptive statistics and correlations: factor analysis (English)*

Variable	*n*	*M*	*SD*	1	2	3	4	5
1. Stress	86	6.23	2.67	--	0.78	0.50	0.56	0.03
2. Worry	86	6.23	2.68		--	0.56	0.59	0.12
3. Lonely	86	5.88	2.71			--	0.62	0.21
4. Change	86	5.78	2.83				--	0.35
5. Family	86	6.98	2.24					--

* French findings in-text.

Principal components analysis was used to assess the factors underlying the 3Cs. Initial eigen values indicated that the first factor explained 57% of the variance, while the second factor explained 21% of the variance. Using varimax and oblimin rotations, the two-factor solution explained 78% of the variance. Specifically, there was a leveling off of eigen values on the scree plot after two factors and there was an insufficient number of primary loadings to interpret three or more factors. A varimax rotation provided the best-defined factor structure, as all items in this analysis had primary loadings over 0.79. Although the French sample size of 25 was much smaller than for the English measure, the factor analysis findings were replicated in French. Results were nearly identical for the French and English measures (French: KMO.68; Bartlett *p* < 0.001; a two-factor solution that explained 80% of the variance; primary loadings over.79).

Composite scores were created for each of the two factors, based on the mean of the items which had their primary loadings on each factor. Higher scores indicate greater use of positive COVID coping. Skewness (−0.13) and kurtosis (−0.53) were found to be within an acceptable range for assuming normal distribution. For the English measure, the mean coping score on the child factor was 24.12 (*sd* = 9.10), range 4 to 40, while the mean coping score on the perceived family coping factor was 6.98 (*sd* = 2.24). Scores below these would suggest poor COVID coping. For the French measure, the mean coping score on the child factor was 26.24 (*sd* = 9.81), range 4 to 40 (skewness −0.75; kurtosis.32), while the mean coping score on the perceived family coping factor was 7.00 (*sd* = 2.46).

### Credibility: Criterion-related validity

Pearson correlations were conducted to determine if the survey factors (child and family) were associated with measures of internalizing and externalizing mental health, as well as meaning mindset. Correlations of the English measures can be found in Table 2 and the French measures can be found in Table 3.

**Table 2:** Correlations between the 3Cs factors, mental health, and meaning mindset (English).

Variable	1	2	3	4
1. 3Cs - child	--	0.21*	−0.50**	0.45**
2. 3Cs - family		--	−0.05	0.15
3. Mental illness symptoms			--	−0.54**
4. Meaning mindset				--

* *p < 0.05;*

** *p* < 0.001.

**Table 3:** Correlations between the 3Cs factors, mental health, and meaning mindset (French).

Variable	1	2	3	4
1. 3Cs - child	--	0.02	−0.54**	0.49**
2. 3Cs - family		--	0.02	0.25
3. Mental illness symptoms			--	−0.73**
4. Meaning mindset				--

** *p* < 0.001.

### Credibility: Reliability

The internal consistency reliability of the English measure (child factor four items) was.86, which is considered to be “good,” based on Hunsley and Mash (2008) criteria. For the French version, internal consistency reliability was similarly “good” at.88 (Hunsley and Mash, 2008).

#### Acceptability

The initial group of mental health practitioners, parents, and five children assessed item acceptability. Specifically, these reviewers were consulted regarding item wording. The mental health practitioners and parents were asked whether children would find each item understandable and whether children might answer it correctly. Each item was perceived as clear, “child friendly,” understandable by the reviewers. Children noted that the items and the measure were easy to complete: “It was fun.”

#### Sustainability

Children thought the measure was fun to complete, but they suggested videos or images could make the measure more fun to want to complete it, for future use: “Maybe there could be pictures or videos to make it more fun, but it was fine as it was.” The mental health practitioners were also asked whether they would be likely to use this measure in their research or in their clinical work. They noted that this measure would be a welcome addition to their clinical practices or their research: “When I’m concerned about how my clients are coping with the pandemic, I think this would be helpful to assess their perceived coping skills.”

#### Feasibility

Knowledge-users stated that the measure in both languages was appropriately brief: “It was a good length. Nice and short.” Through online survey statistics, average completion time was also recorded (approximately 1 min). Further, response rates and missing values analyses were conducted. For example, for the English measure, those who completed the measure completed 100% of the measure. Out of 97 original English participants, 86 completed the measure, while 11 did not complete any questions after completing the criterion-related validity questionnaires (including those who completed some parts of those questionnaires or simply the informed consent checkbox).

## Discussion

The current study contributes a new measure to the literature, allowing for children’s voices to be heard during the pandemic, during future pandemics, or during other major life-changing societal events. To date, it is the first brief measure of pandemic-specific, or societal disruption crisis-specific coping, addressing the domains of coping with stress, worries, loneliness, and school changes, as well as family coping. This measure should hopefully ease the evaluation of mental health promotion programming to enhance child well-being during this time period and beyond.

Through a KTI model, children, parents, and mental health practitioners were collaboratively engaged in measure development. This study yielded a valid four-item measure of child pandemic coping in both English and French. Given that the fifth family coping item did not display criterion-related validity with child mental health and meaning mindset, it is possible that this item may need to be reworded (e.g., to parental coping, rather than family coping). Previous literature suggests that poor parental coping is predictive of poorer child mental health outcomes (Mochida et al., 2021). This study also yielded mean scores to determine what would reflect “poor coping,” one standard deviation below the mean: A score below 15 would generally reflect poor pandemic coping.

### Knowledge translation-informed measure development

As in our past measure and program development research, borrowing from the scientific utility standards, we aimed to develop a measure that is credible, acceptable, sustainable, and feasible (Joint Committee on Standards for Educational Evaluation, 1981; Judd et al., 2001). Knowledge-users, including children, parents, and mental health practitioners—anyone for whom the use of this measure would affect—were co-creators in the design of the 3Cs. Engaging stakeholders can result in a measure that is a better fit for them, as well as generate more “buy-in” through collaboration for use of the measure in research and practice (Armstrong and Watt, 2020).

#### Credibility

Through collaboration, knowledge-users noted that the 3Cs was both content and face valid. Specifically, they indicated that it reflected appropriate literature-based domains representing COVID-coping. Internal consistency reliability of both the English and French version of the measure were comparably good. Further, criterion-related validity of the measure was established with related concepts: Mental health and meaning mindset. Further, although not part of the scope of the present study, but as part of a larger study (Potter, 2022), measure sensitivity to change was also noted. Following the implementation of a mental health promotion program teaching skills to address agency over thoughts and behaviour, social–emotional literacy, and meaningful engagement, 3Cs scores were significantly enhanced from pretest (M = 20.5, *sd* = 9.7) to posttest (M = 29.1, *sd* = 6.4).

#### Acceptability, sustainability, and feasibility

The four-item final 3Cs was perceived by children, parents, and mental health practitioners to be acceptably child friendly, worded appropriately, and feasible for use with its brief length. It was further perceived by mental health practitioners to be a useful measure for psychotherapy clinical assessment of coping or for use in research and program evaluation.

### Convergent validity

#### Mental health

As expected, the four-item 3Cs was associated with general mental health (internalizing and externalizing). Thus, children who perceive themselves to have good coping skills regarding changes associated with a major event, such as a pandemic, also reported positive mental health. Specifically, children who noted that they would be able to cope with stress, worries, loneliness, and school changes associated with the pandemic also reported positive mental health. This means that, if intrapersonal, interpersonal, and behavioural mental health is promoted, then children may cope well with major stressors, such as a pandemic. Similarly, if coping strategies were to be taught to manage stress, worries, social connectedness, and life changes (e.g., school changes that occur during a pandemic), then children may be resilient regarding their mental health. Overall, given the associations between pandemic coping and mental health, the 3Cs demonstrated good convergent validity.

#### Meaning mindset

In addition to the association between the 3Cs and mental health, the 3Cs measure was also associated with meaning mindset. Specifically, perceived ability to cope with stress, worries, social disconnection, and changes to important things such as school, were associated with choice and responsibility over thoughts, feelings, and behavior, positive self-concept, hope for the future, and openness to experience. Therefore, just as a meaning mindset is predictive of mental health (Arslan and Yildirim, 2021; Parrot et al., 2021), cultivating a meaning mindset likely also leads to positive coping with difficult circumstances (Frankl, 1986). Further, from a SWPP perspective, there is a dialectical nature to flourishing (Lomas and Ivtzan, 2016). Thus, positively coping with difficult circumstances, moving beyond simply surviving or existing, likely also allows for children to perceive meaning. Regardless on the direction of the correlation, or whether it is bidirectional, given the association between meaning mindset and the 3Cs, this measure demonstrated the expected convergent validity.

### Limitations

To carry out a Factor Analysis, a minimum sample size should be five participants for each item (Garson, 2008). For the current study, a minimum sample of 25 participants was required for each language group. This requirement was met, with 86 participants completing the English measure and 25 completing the French measure. However, in the majority of validation studies, sample sizes are often over 100 (Anthoine et al., 2014). The current sample size was smaller than this value, when divided by language group. Therefore, future research should be conducted with a larger sample. Further, a more even sample of English and French participants would have been ideal. In addition, future validation studies should also include gender and age difference explorations. A parent version of the measure in the future may also serve to provide a multi-informant perspective on child coping, as well as allow for the exploration of interrater reliability. As the present research was a pilot study exploring the use of this measure with a sample of children experiencing social and academic disruptions as a result of the pandemic, further research should consider validating this flexible measure with other samples of children experiencing societal disruption crises (e.g., children displaced from their social and educational circles by war).

### Implications

Given that brief measures of coping with major world events, such as a global pandemic, from a child perspective are not widely available, the development of this new measure fills this significant gap. This new measure allows for the potential wide-scale collection of epidemiological coping data, as well as the evaluation of mental health promotion and therapy approaches during such a time period as a pandemic. This measure could also be explored with other child populations, such as those coping with displacement as a result of war. Epidemiological data from a child perspective would be vital during major world events in order to know how to best meet their needs and enhance their meaning and well-being (Waddell et al., 2013; Parrot et al., 2021). If the voice of children is ignored when decisions are made about them or for them, then resulting programs and services can fail to meet their needs. In a situation where children will most likely live through future pandemics and major global events, children should be engaged in the decision-making process about issues affecting them, potentially resulting in better coping, mental health, and meaning mindset outcomes. Further, given the association between meaning mindset and positive coping during times of global crises, SWPP mental health promotion programming aiming to enhance meaning mindset through the C.H.A.N.G.E. framework noted previously may ultimately lead to good child coping, as well as mental health.

## Data availability statement

The raw data supporting the conclusions of this article will be made available by the authors, without undue reservation.

## Ethics statement

The studies involving human participants were reviewed and approved by Saint Paul University Ethical Review Board. Written informed consent to participate in this study was provided by the participants’ legal guardian/next of kin.

## Author contributions

LA and CP (MA thesis student) collaborated on this publication. LA was the primary author of the article, while CP was the primary collector of research data and contributed to the writing of the article. All authors contributed to the article and approved the submitted version.

## Conflict of interest

The authors declare that the research was conducted in the absence of any commercial or financial relationships that could be construed as a potential conflict of interest.

## Publisher’s note

All claims expressed in this article are solely those of the authors and do not necessarily represent those of their affiliated organizations, or those of the publisher, the editors and the reviewers. Any product that may be evaluated in this article, or claim that may be made by its manufacturer, is not guaranteed or endorsed by the publisher.

## Footnotes

^1^Insert major event here (i.e., COVID-19 pandemic).

## References

[ref1] Abawi O. Welling M. S. van den Eynde E. van Rossum E. F. C. Halberstadt J. van den Akker E. L. T.. (2020). COVID-19 related anxiety in children and adolescents with severe obesity: a mixed methods study. Clin. Obe. 10:e12412. doi: 10.1111/cob.12412, PMID: 32920993 PMC7685119

[ref2] American Psychological Association. (1985). Standards for educational and psychological testing. Washington: American Psychological Association.

[ref3] Amsden J. VanWynsberghe R. (2005). Community mapping as a research tool with youth. Action Res. 3, 357–381. doi: 10.1177/1476750305058487

[ref4] Anthoine E. Moret L. Regnault A. Sébille V. Hardouin J. B. (2014). Sample size used to validate a scale: a review of publications on newly-developed patient reported outcomes measures. Health Qual. Life Outcomes 12:176. doi: 10.1186/s12955-014-0176-2, PMID: 25492701 PMC4275948

[ref5] Armstrong L. L. Desson S. St. John E. Watt E. (2018). The D.R.E.A.M. Program: Developing Resilience through Emotions, Attitudes, & Meaning (Gifted Edition) – Second Wave Positive Psychology Approach. Couns. Psychol. Q. 32, 307–332. doi: 10.1080/09515070.2018.1559798

[ref6] Armstrong L.L. Watt E., St. John, E., & Desson (2020). The interactive symptoms assessment: I.S.a. – development and validation using a knowledge translation-integrated model. Curr. Psychol. doi:10.1007/s12144-020-00801-5, 41, 3038, 3054

[ref7] Armstrong L. L. Watt E. St. John, E., & Desson, S. (2019). The child identity and purpose questionnaire–interactive: development and validation of the revised, video-based version using a knowledge translation-integrated approach. Humanist. Psychol. 48, 298–317. doi: 10.1037/hum0000147

[ref8] Arslan G. Yildirim M. (2021). Coronavirus stress, meaningful living, optimism, and depressive symptoms: a study of moderated mediation model. Aust. J. Psychol. 73, 113–124. doi: 10.1080/00049530.2021.1882273

[ref9] Barblett L. Maloney C. (2010). Complexities of assessing social and emotional competence and wellbeing in young children. Aust. J. Early Child. 35, 13–18. doi: 10.1177/183693911003500203

[ref10] Bouwmans C. De Jong K. Timman R. Zijlstra-Vlasveld M. Van der Feltz-Cornelis C. Tan Swan S.. (2013). Feasibility, reliability and validity of a questionnaire on healthcare consumption and productivity loss in patients with a psychiatric disorder (TiC-P). BMC Health Serv. Res. 13, 213–217. doi: 10.1186/1472-6963-13-217, PMID: 23768141 PMC3694473

[ref11] Brancato G. Macchia S. Murgia M. Signore M. Simeoni G. Blanke K. Hoffmeyer-Zlotnik J. (2006). Handbook of recommended practices for questionnaire development and testing in the European statistical system. European Statistical System.

[ref12] Clemens V. Deschamps P. Fegert J. Anagnostopoulos D. Bailey S. Doyle M.. (2020). Potential effects of “social” distancing measures and school lockdown on child and adolescent mental health. Eur. Child Adolesc. Psychiatry 29, 739–742. doi: 10.1007/s00787-020-01549-w, PMID: 32447569 PMC7245163

[ref13] Cohen S. R. Mount B. M. Strobel M. G. Bui F. (1995). The McGill quality of life questionnaire: a measure of quality of life appropriate for people with advanced disease. A preliminary study of validity and acceptability. Palliat. Med. 9, 207–219. doi: 10.1177/026921639500900306, PMID: 7582177

[ref14] Commissioner for Children and Young People. (2016). Involving children and young people: Participation guidelines. Western Australia: Commissioner for Children and Young People. Available at: https://www.ccyp.wa.gov.au/media/1463/report-our-children-cant-wait-december-2015.pdf

[ref15] Cooper K. Hards E. Moltrecht B. Reynolds S. Shum A. McElroy E.. (2021). Loneliness, social relationships, and mental health in adolescents during the COVID-19 pandemic. J. Affect. Disord. 289, 98–104. doi: 10.1016/j.jad.2021.04.016, PMID: 33962368 PMC9310699

[ref16] Cost K. T. Crosbie J. Anagnostou E. Birken C. S. Charach A. Monga S.. (2021). Mostly worse, occasionally better: impact of COVID-19 pandemic on the mental health of Canadian children and adolescents. Europ. Child Adol. Psychiatry 31, 671–684. doi: 10.1007/s00787-021-01744-3, PMID: 33638005 PMC7909377

[ref17] de Avila G. Andréia M. da Silva Jacob F. L. Alcantara L. R. S. Berghammer M. Nolbris M. J.. (2020). Children’s anxiety and factors related to the COVID-19 pandemic: an exploratory study ising the Children’s anxiety questionnaire and the numerical rating scale. Int. J. Environ. Res. Public Health 17:5757. doi: 10.3390/ijerph17165757, PMID: 32784898 PMC7459447

[ref18] Deighton J. Courdace T. Fonagy P. Brown J. Patalay P. Wolpert W. (2014). Measuring mental health and well-being outcomes for children and adolescents to inform practice and policy: a review of child self-report measures. Child Adolesc. Psychiatry Ment. Health 8, 1–14. doi: 10.1186/1753-2000-8-14, PMID: 24834111 PMC4022575

[ref19] Dweck C. S. (2015). Growth [editorial]. Br. J. Educ. Psychol. 85, 242–245. doi: 10.1111/bjep.12072 25973689

[ref20] Fegert J. M. Vitiello B. Plener P. L. Clemens V. (2020). Challenges and burden of the coronavirus 2019 (COVID-19) pandemic for child and adolescent mental health: a narrative review to highlight clinical and research needs in the acute phase and the long return to normality. Child Adolesc. Psychiatry Ment. Health 14:20. doi: 10.1186/s13034-020-00329-3, PMID: 32419840 PMC7216870

[ref21] Fineberg N. A. Van Ameringen M. Drummond L. Hollander E. Stein D. J. Geller D.. (2020). How to manage obsessive-compulsive disorder (OCD) under COVID-19: a clinician's guide from the International College of Obsessive Compulsive Spectrum Disorders (ICOCS) and the obsessive-compulsive and related disorders research network (OCRN) of the European College of Neuropsychopharmacology. Compr. Psychiatry 100:152174. doi: 10.1016/j.comppsych.2020.152174, PMID: 32388123 PMC7152877

[ref22] Frankl V. (1986). Man’s search for meaning. New York: Simon & Schuster.

[ref23] Garson D.G. (2008) Factor analysis: Statnotes. Available at: http://www2.chass.ncsu.edu/garson/pa765/factor.htm (Accessed March 2022).

[ref24] Gough Kenyon S. M. Palikara O. Lucas R. M. (2021). Consistency of parental and self-reported adolescent wellbeing: evidence from developmental language disorder. Front. Psychol. 12:629577. doi: 10.3389/fpsyg.2021.629577, PMID: 33776852 PMC7991577

[ref25] Greene R. W. Ablon J. S. (2006). Treating explosive kids: The collaborative problem-solving approach. New York: Guilford Press.

[ref26] Haig-Ferguson A. Cooper K. Cartwright E. Loades M. Daniels J. (2021). Practitioner review: health anxiety in children and young people in the context of the COVID-19 pandemic. Behav. Cogn. Psychother. 49, 129–143. doi: 10.1017/S1352465820000636, PMID: 32829718 PMC7503041

[ref27] Hunsley J. Mash E. J. (2008). “Developing criteria for evidence-based assessment: an introduction to assessments that works,” in A guide to assessments that work. eds. Hunsley J. Mash E. J. (New York: Oxford University Press), 3–14.

[ref28] Husky M. M. Boyd A. Bitfoi A. Carta M. G. Chan-Chee C. Goelitz D.. (2018). Self-reported mental health in children ages 6-12 years across eight European countries. Eur. Child Adolesc. Psychiatry 27, 785–795. doi: 10.1007/s00787-017-1073-0, PMID: 29082450

[ref29] International Organization for Standardization (2017). Sustainability. Available at: https://www.iso.org (Accessed March 2022).

[ref59] Ivtzan I. Lomas T. Hefferon K. Worth P. (2016). *Second Wave Positive Psychology: Embracing the Dark Side of life.* London, UK: Taylor & Francis Ltd.

[ref30] Janssens J. J. Achterhof R. Lafit G. Bamps E. Hagemann N. Hiekkaranta A. P.. (2021). The impact of COVID-19 on adolescents’ daily lives: the role of parent–child relationship quality. J. Res. Adolesc. 31, 623–644. doi: 10.1111/jora.12657, PMID: 34448305 PMC8646476

[ref31] Joint Committee on Standards for Educational Evaluation. (1981). Standards for Evaluations of Educational Programs, Projects, and Materials. New York: Mc-Graw Hill Company.

[ref32] Judd J. Frankish J. Moulton G. (2001). Setting standards in the evaluation of community-based health promotion programmes—a unifying approach. Oxf. J. Med. Health Promotion Int. 16, 367–380. doi: 10.1093/heapro/16.4.367 11733455

[ref33] Kar S. K. Yasir Arafat S. M. Kabir R. Sharma P. Saxena S. K. (2020). “Coping with mental health challenges during COVID-19” in Coronavirus disease 2019 (COVID-19). Medical virology: From pathogenesis to disease control. ed. Saxena S. (Singapore: Springer).

[ref34] Kroesbergen E. H. van Hooijdonk M. Van Viersen S. Middel-Lalleman M. M. N. Reijnders J. J. W. (2016). The psychological well-being of early identified gifted children. Gifted Child Q. 60, 16–30. doi: 10.1177/0016986215609113

[ref35] Liratni M. Pry R. (2011). Enfants à haut potentiel intellectuel: Psychopathologie, socialisation et comportements adaptatifs. Neuropsychiatr. Enfance Adolesc. 59, 327–335. doi: 10.1016/j.neurenf.2010.11.005

[ref36] Liu C. H. Doan S. N. (2020). Psychosocial stress contagion in children and families during the COVID-19 pandemic. Clin. Pediatr. 59, 853–855. doi: 10.1177/0009922820927044, PMID: 32462929

[ref37] Lomas T. Ivtzan I. (2016). Second wave positive psychology: exploring the positive–negative dialectics of wellbeing. J. Happiness Stud. 17, 1753–1768. doi: 10.1007/s10902-015-9668-y

[ref38] Mochida S. Sanada M. Shao Q. Lee J. Takaoka J. Ando S.. (2021). Factors modifying children’s stress during the COVID-19 pandemic in Japan. Eur. Early Child. Educ. Res. J. 29, 51–65. doi: 10.1080/1350293X.2021.1872669

[ref39] Mohler-Kuo M. Dzemaili S. Foster S. Werlen L. Walitza S. (2021). Stress and mental health among children/adolescents, their parents, and young adults during the first COVID-19 lockdown in Switzerland. Int. J. Environ. Res. Public Health 18:4668. doi: 10.3390/ijerph18094668, PMID: 33925743 PMC8124779

[ref40] Munir F. (2021). Mitigating COVID: impact of COVID-19 lockdown and school closure on children’s well-being. Soc. Sci. 10:387. doi: 10.3390/socsci10100387

[ref41] Nietzsche F. (1889, 2003) Twilight of the idols: Or, how to philosophize with the hammer (Trans. Polt, R). Indianapolis: Hackett Publishing Company.

[ref42] Parrot J. Armstrong L. L. Watt E. Fabes R. Timlin R. (2021). Building resilience during COVID-19: recommendations for adapting the DREAM program – live edition to an online-live hybrid model for in-person and virtual classrooms. Front. Psychol. Edu. Psychol. 12. doi: 10.3389/fpsyg.2021.647420, PMID: 34322055 PMC8311233

[ref43] Pilarinos V. Solomon C. R. (2017). Parenting styles and adjustment in gifted children. Gifted Child Q. 61, 87–98. doi: 10.1177/0016986216675351

[ref44] Potter C. (2022). A knowledge translation-integrated approach: Evaluating the DREAM Program’s webisodes and French translation (Master’s thesis). Ottawa: Saint Paul University.

[ref45] Riley A. (2004). Evidence that school-age children can self-report on their health. Ambul. Pediatr. 4, 371–376. doi: 10.1367/A03-178R.1 15264962

[ref46] Sarıalioğlu A. Atay T. Arıkan D. (2022). Determining the relationship between loneliness and internet addiction among adolescents during the covid-19 pandemic in Turkey. J. Pediatr. Nurs. 63, 117–124. doi: 10.1016/j.pedn.2021.11.011, PMID: 34801327 PMC8916416

[ref47] Shah K. Kamrai D. Mekala H. Mann B. Desai K. Patel R. S. (2020). Focus on mental health during the coronavirus (COVID-19) pandemic: applying learnings from the past outbreaks. Cureusm 12:e7405. doi: 10.7759/cureus.7405, PMID: 32337131 PMC7182052

[ref48] Shantall T. (1989). “Viktor Frankl’s existential theory” in Personality Theories – From Freud to Frankl. eds. Meyer W. F. Moore C. Viljoen H. G. (Johannesburg: Lexicon), 421–439.

[ref49] St. John E. (2017). Meaning as an early determinant of childhood mental health: potential influence of religious attendance (Master’s thesis). Retrieved from University of Ottawa database.

[ref50] Stoddard S. A. Henly S. J. Sieving R. E. Bolland J. (2011). Social connections, trajectories of hopelessness and serious violence in impoverished urban youth. J. Youth Adol. 40, 278–295. doi: 10.1007/s/10964-010-9580-z, PMID: 20690037 PMC3105375

[ref51] Taylor S. (2019). Preparing for the next global outbreak of infectious disease. Cambridge: Cambridge Scholars Publishing.

[ref52] Trochim W. Donnelly J. P. Arora K. (2016). Research methods: The essential knowledge base (2nd ed.). California: Wadsworth Publishing.

[ref53] Ullah I. Razzaq A. De Berardis D. Ori D. Adiukwu F. Shoib S. (2021). Mental health problems in children & pandemic: dangers lurking around the corner and possible management. Int.l J. Soc. Psychiatry 68, 693–696. doi: 10.1177/0020764021992816 33554702

[ref54] Van Tongeren D. R. Green J. D. (2010). Combating meaninglessness: on the automatic defense of meaning. Personal. Soc. Psychol. Bull. 36, 1372–1384. doi: 10.1177/0146167210383043, PMID: 20805340

[ref55] Waddell C. Shepherd C. A. Chen A. Boyle M. H. (2013). Creating comprehensive children’s mental health indicators for British Columbia. Can. J. Commun. Ment. Health 32, 9–27. doi: 10.7870/cjcmh-2013-003

[ref56] Watt E. (2020). Dreaming of a solution: D.R.E.A.M.-O.F. a mental health promotion program for children and their families on mental health waitlists. Doctoral dissertation. Ottawa: Saint Paul University.

[ref60] Wong P. T. P. (2011). Positive psychology 2.0: Towards a balanced interactive model of the good life. Canadian Psychology Psychologie Canadienne 52, 69–81.

[ref57] Wong P. (2017). Meaning-centred approach to research and therapy, second wave positive psychology, and the future of humanistic psychology. Humanist. Psychol. 45, 207–216. doi: 10.1037/hum0000062

[ref61] Wong P. T. P. Worth P. (2017). The deep-and-wide hypothesis in giftedness and creativity [Special issue]. Psychology and Education, 54. Available at: http://www.psychologyandeducation.net/pae/category/volume-54-no-3-4-2017/

